# The Effects of CPAP Treatment on Task Positive and Default Mode Networks in Obstructive Sleep Apnea Patients: An fMRI Study

**DOI:** 10.1371/journal.pone.0047433

**Published:** 2012-12-05

**Authors:** Olga Prilipko, Nelly Huynh, Sophie Schwartz, Visasiri Tantrakul, Clete Kushida, Teresa Paiva, Christian Guilleminault

**Affiliations:** 1 Stanford University Sleep Clinic and Center for Human Sleep Research, Redwood City, California, United States of America; 2 Geneva University Medical Center, Geneva, Switzerland; 3 Department of Neurology, CENC, Lisbon, Portugal; The Chinese University of Hong Kong, Hong Kong

## Abstract

**Introduction:**

Functional magnetic resonance imaging studies enable the investigation of neural correlates underlying behavioral performance. We investigate the effect of active and sham Continuous Positive Airway Pressure (CPAP) treatment on working memory function of patients with Obstructive Sleep Apnea Syndrome (OSAS) considering Task Positive and Default Mode networks (TPN and DMN).

**Methods:**

An experiment with 4 levels of visuospatial n-back task was used to investigate the pattern of cortical activation in 17 men with moderate or severe OSAS before and after 2 months of therapeutic (active) or sub-therapeutic (sham) CPAP treatment.

**Results:**

Patients with untreated OSAS had significantly less deactivation in the temporal regions of the DMN as compared to healthy controls, but activation within TPN regions was comparatively relatively preserved. After 2 months of treatment, active and sham CPAP groups exhibited opposite trends of cerebral activation and deactivation. After treatment, the active CPAP group demonstrated an increase of cerebral activation in the TPN at all task levels and of task-related cerebral deactivation in the anterior midline and medial temporal regions of the DMN at the 3-back level, associated with a significant improvement of behavioral performance, whereas the sham CPAP group exhibited less deactivation in the temporal regions of Default Mode Network and less Task Positive Network activation associated to longer response times at the 3-back.

**Conclusion:**

OSAS has a significant negative impact primarily on task-related DMN deactivation, particularly in the medial temporal regions, possibly due to nocturnal hypoxemia, as well as TPN activation, particularly in the right ventral fronto-parietal network. After 2 months of active nasal CPAP treatment a positive response was noted in both TPN and DMN but without compete recovery of existing behavioral and neuronal deficits. Initiation of CPAP treatment early in the course of the disease may prevent or slow down the occurrence of irreversible impairment.

## Introduction

Obstructive sleep apnea syndrome (OSAS) results in recurrent hypoxic episodes during sleep, fragmented sleep, cardiovascular comorbidities, neurocognitive impairment during the day, and excessive daytime sleepiness. Aspects of executive functions are consistently found impaired in behavioral studies on the impact of OSAS on cognitive function, but response to Continuous Positive Airway Pressure (CPAP) treatment varies [1], [2]. It has been proposed that executive deficits are primarily related to the severity of nocturnal hypoxemia and therefore reflect an irreversible damage of the brain tissue, hence showing moderate or low response to CPAP treatment [3], [4], [5], [6].

With the advent of non-invasive neuroimaging techniques, such as functional magnetic resonance imaging (fMRI), it is now possible to examine the neural correlates of cognitive performance as well as the individual impact of OSAS-related variables such as nocturnal hypoxemia and sleep fragmentation on brain function. Several fMRI studies conducted previously have reported both increased or decreased brain activation in OSAS patients. These changes have been attributed to either a compensatory recruitment of brain resources in order to maintain performance or to brain damage secondary to nocturnal hypoxemia respectively [7], [8], [9], [10], [11], [12]. Most studies have focused on changes of cerebral activation within brain regions that consistently exhibit an increase of activation with tasks requiring attention and/or executive control. The network of those regions has been previously described as Task Positive Network (TPN) as it exhibits an increase in activation when subjects engage in cognitive tasks. TPN comprises several functional networks reported in cognitive literature such as the fronto-parietal attention network, executive network and right hemisphere-lateralized ventral frontal networks [13], [14].

In the recent years however, it has become clear that a set of brain regions operates antagonistically to the TPN and that optimal coupling between the TPN and this set is mandatory for successful behavioral performance [15], [16]. This network of TPN-anticorrelated brain regions is more active during rest and responds with progressive deactivation to external goal-oriented task. It has been described and is commonly referred to as Default Mode Network (DMN) in studies of functional connectivity or Task Negative Network/Task Deactivation Network (TNN/TDN) in studies of cognitive task performance [16], [17], [18]. In this report we shall refer to the network of regions that exhibit progressive n-back related deactivation as “DMN”.

Examining these networks in a previous study we found that OSAS had a negative impact on both TPN and DMN [19]. Therefore the aim of this study was to examine 1) the effects of CPAP versus sham-CPAP treatment on behavioral performance and brain function in both networks in OSAS patients and 2) to compare the activation and deactivation patterns to those of healthy controls (HC).

Based on previous literature, we hypothesize that:

- CPAP treatment would result in a decrease of compensatory recruitment within the TPN and a normalization of the DMN deactivation pattern in OSAS patients whose baseline behavioral performance did not differ from that of HC;
- An increase of compensatory recruitment of cerebral regions adjacent to TPN/DMN would be noted in OSAS patients whose baseline behavioral performance was lower than that of HC, but who improved with treatment.

## Methods

### Subjects

Seventeen men diagnosed with moderate to severe OSAS were recruited from the Stanford Sleep Clinic and surrounding area via advertisement. All participants (patients and healthy controls) were right-handed nonsmokers and were screened for current or previous neurological and psychiatric disorder as determined by history, clinical evaluation, and Hamilton Depression Scale score. All participants reported regular sleep schedules with ≥6 h of sleep per night as determined by sleep habits questionnaires. Seven age-matched subjects without history of sleep disorders were recruited from the community as healthy controls (HC). Absence of sleep pathology in HC, including sleep disordered breathing (SDB), was confirmed by an overnight polysomnography (PSG: AHI <5).

After a baseline fMRI scan, patients were randomly assigned to either the active (therapeutic, n = 9) or sham (sub-therapeutic, n = 8) nasal CPAP group. A CPAP titration study was conducted for all patients in both groups: the “active group” subjects were effectively titrated to the appropriate nasal CPAP pressure whereas the “sham group” subjects slept with the sub-therapeutic nasal CPAP previously used in sham CPAP studies [20]. The sham-CPAP device closely simulated the airflow through the exhalation port and the operating noise of the active-CPAP device. At the end of the treatment period, subjects in the sham-CPAP group underwent a second CPAP titration night and left the study with therapeutic CPAP treatment. Prior study using a functionally similar sham-CPAP device revealed that oxygen saturation, end-tidal CO2, and mean temperature and humidity measured at the CPAP mask are the same with the active and the sham-CPAP [20]. And after 2 months, with treatment compliance monitored using the Encore® Pro Smart Card® system, the comparison of sham CPAP subjects to a no treatment group with 10 OSAS men matched for age and AHI, showed no significant difference in sleep parameters or number of abnormal respiratory events [20].

Previous studies have shown improvement in cognitive function in OSAS patients after as little as 2 weeks of CPAP treatment and therefore we chose the 2-month treatment period in our study as an optimal duration in regard to active and sham-CPAP compliance.

The study was approved by the Stanford Institutional Review Board, and all subjects signed informed consent.

### Polysomnography (PSG)

All subjects had overnight PSGs. The following variables were systematically monitored: EEG, electro-oculogram, electrocardiogram, chin and leg myogram, nasal air flow with nasal cannula, abdominal and thoracic respiratory movements with inductive respiratory belts, and pulse oximetry. Studies were scored by independent technicians and reviewed by a qualified sleep medicine physician according to the AASM scoring criteria.

### Experimental Procedure and n-Back Task

Subjects were instructed to abstain from ingestion of any caffeinated beverages ≥9 h prior to scanning. Scanning was performed at same evening time for all subjects and both sessions (before and after treatment). Healthy controls were scanned only once (baseline).

During the fMRI session, participants performed 2 sessions of visuo-spatial n-back task generated with E-Prime 1.0 software and projected on a mirror in front of the subject's eyes. Participants performed two 9 min 40 sec sessions of a block-designed parametric n-back task with 4 levels of difficulty (0-back, 1-back, 2-back, and 3-back). Each session comprised 2 blocks of each WM condition (1-, 2-, and 3-back) of 63 sec duration, separated by 5 blocks of the baseline condition (0-back) of 25.2 sec duration each (0-1-0-2-0-3-0-1-0-2-0-3). During the task, a white dot flashed for 200 ms in 6 possible locations on a black screen, in a pseudo-random order. Fifty percent of stimuli were matches.

Subjects were instructed to respond as fast and as accurately as possible with their right hand (index finger for matches and middle finger for mismatch). In 0-back condition they had to indicate whether the dot appeared on the left or on the right side of the screen, or whether each given dot appeared in the same or different location as the dot that was flashed 1-,2-, or 3-dots before (in 1-, 2-, and 3-back conditions, respectively). Subjects' responses during the n-back sessions and their response times (RT) in the scan were recorded via a custom-made response box in the E-Prime Data Aid software. In order to minimize learning effects and maximize behavioral performance subjects were trained on the task until they reached a minimum of 75% of correct responses during the week preceding the first fMRI session and again immediately before the first and second fMRI sessions.

### fMRI Data Acquisition

Functional MRI data was acquired on a 3.0T GE (Milwaukee, WI) whole-body scanner with a custom quadrature bird-cage head coil. Head movement was minimized with foam padding. Thirty axial slices were taken parallel to the anterior/posterior commissure plane (AC-PC) with 4-mm slice thickness, 1-mm interslice gap. High- esolutionT2 weighted fast spin echo structural images were acquired for anatomical reference. A T2*-sensitive gradient echo spiral in/out pulse sequence [21] was used for functional imaging (TR  = 2000 ms, TE  = 30 msec, flip angle  = 70, FOV  = 24 cm, matrix  = 64×64). An automated high-order shimming procedure based on spiral acquisitions was used to reduce B0 heterogeneity [22]. A high-resolution T1 volume scan (124 slices, 1.2-mm thickness) was collected for every subject using an IR-prep FSPGR sequence for T1 contrast.

### fMRI Data Analysis

Functional MRI data were preprocessed and analyzed using the Statistical Parametric Mapping software (SPM; Welcome Department of Cognitive Neurology, London) and the custom MATLAB routines (MathWorks Natick, MA). The preprocessing steps consisted of realignment of all images to the first image, normalization to MNI template, and spatial smoothing with a Gaussian filter of 6 mm full-width-half-maximum.

To test for the effect of each task load, we used a standard general linear approach with 4 regressors for the 3 task load and baseline conditions, modeled as a boxcar function convolved with the canonical HRF. The 6 motion parameters from the realignment were added as 6 regressors of no interest. Statistical analysis at the single-subject level treated each voxel according to a general linear model [23]. Individual contrast images were created by computing each WM task load versus the 0-back load baseline (1>0, 2>0, 3>0). For group analysis, paired t-tests (baseline versus post-treatement) were performed for the 3 main contrasts (1vs0, 2vs0 and 3vs0) in both therapeutic and sham-CPAP groups in order to assess within group changes and a regressor of no-interest was included in the analysis to account for CPAP compliance (% of nights with >4 h CPAP use). A flexible factorial design was used to examine treatment group versus session interaction for the 3 main contrasts (1vs0, 2vs0 and 3vs0) in order to assess between-group differences. Analysis was confined to either a TPN (size: 9937mm3) or a DMN mask (size: 36535mm3), constructed from TPN and DMN activation/deactivation maps of HC in the 3vs0-back contrast.

**Figure 1:**
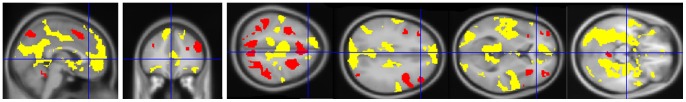
TPN (red) and DMN (yellow) masks derived from 3vs0 contrast task-related activation and deactivation respectively of HC (corrected for multiple comparisons, p = 0.05, presented in neurological convention).

The between-condition statistical threshold was set to (p<0.01 at voxel level, uncorrected) and cluster size >42 voxels for TPN (|t|>3 for 1vs0 and 2vs0-back and |t|>2.9 for 3vs0-back for the active-CPAP group) and (p<0.01 at voxel level, uncorrected) cluster size>58 voxels for DMN masks (|t|>3.14 fro 2vs0 and 3vs0-back for sham-CPAP and |t|>2.9 for 3vs0-back for the active-CPAP groups), which corresponds to multiple comparison corrected p<0.05 (Fig. 1). These corrections were determined by Monte-Carlo simulations performed in REST software [24], [25].

For comparison between each patient group (active and sham) and HC, two-way ANOVAs were performed for the 3 main contrasts (1vs0, 2vs0 and 3vs0). Analysis was confined to either a TPN or a DMN mask (see above). Additionally, an inclusive masking procedure was performed for TPN activation (3vs0-back contrast) and DMN deactivation (0vs3-back contrast) for patients and HC at baseline and after treatment to reveal voxels significantly activated in one and the other (inclusive) groups. SPM inclusive masks were thresholded at P<0.05 uncorrected, whereas the contrasts to be masked were thresholded using a stringent FDR correction for multiple comparisons at p<0.01 for 0vs3-back contrast in the sham-CPAP and HC groups, and at p<0.05 for all other conditions. Inclusive masking analysis allowed us to investigate differences in activation and deactivation between OSAS patients and HC.

Finally, a multiple regression analysis was performed in order to test for correlation of task load effect on cerebral activation/deactivation within TPN and DMN with clinical measures such as duration of nocturnal hypoxemia and AHI. To that effect we used a parametric model where all task blocks were modelled as one single regressor, with 2 additional regressors modeling a linear modulation of the task-related activity by load level (1-, 2-, and 3-back), a quadratic modulation of the task-related activity by load level, and 7 regressors of no interest (behavioral performance per task block and 6 motion correction parameters). Contrast images generated by the linear load regressor from each subject entered the multiple regression group analysis with time spent under 90% SpO_2_ (minutes) and AHI as covariates. Analysis was confined to either a TPN or a DMN mask (see above).

## Results

### Clinical Measure and Behavioral Performance

#### 1. Baseline patient data and treatment compliance

In spite of double-blind randomization procedure, the CPAP group had significantly higher BMI (p = 0.02) than the sham-CPAP group and tended to be more affected by OSAS as reflected by the PSG variables, even though the trend did not reach significance due to the large standard deviations present which was related to the limited number of studied subjects (Table 1).

**Table 1:** Sample characteristics.

	Active-CPAP	Sham-CPAP	p-value
Age	44.7 (+/−8.8)	41.6 (+/−8.3)	0.5
BMI	29.9 (+/−4.2)	25.5 (+/−2.1)	**0.02**
AHI	45.8 (+/−26)	32.8 (+/−17.7)	0.2
ESS	6.8 (+/−5.5)	9 (+/−3.7)	0.3
TST	371.4 (+/−85.7)	369.8 (+/−45.4)	1
Sleep efficiency	82.2 (+/−16.3)	83.6 (+/−9.3)	0.8
REM	17.2 (+/−4.9)	15.7 (+/−6.9)	0.6
Stage 1	12.1 (+/−9)	10.1 (+/−6.6)	0.6
Stage 3+4	5.8 (+/−7.2)	8 (+/−9)	0.6
Minutes <90% SpO_2_	75.4 (+/−123.3)	6.6 (+/−8)	0.1
Treatment compliance	59 (+/−29)	69 (+/−30)	0.6
(% of days with >4h CPAP use)			

BMI  =  body mass index, AHI  =  apnea hypopnea index, ESS  =  Epworth Sleepiness Scale, TST  =  total sleep time, REM  =  rapid eye movement sleep.

Active and sham-CPAP groups had 59% (+/−29) and 69% (+/−30) of total treatment nights with >4 h of CPAP use respectively.

#### 2. Behavioral performance at baseline and post-treatment

During scanning, patients' accuracy decreased and response time increased with increasing n-back load. At baseline, a two-way ANOVA showed a significant group × n-back interaction for HC, active and sham CPAP groups for performance accuracy (p = 0.023). A subsequent one-way ANOVAs showed that active CPAP OSA patients' group performed significantly worse than HC on the 3-back, both before (75.2% vs 89% correct, p = 0.002) and after CPAP treatment (78.7% versus 89% correct, p = 0.04) (Table 2).

**Table 2:** Behavioral performance.

	Sham-CPAP group		Actif-CPAP group		HC	Sham vs HC	Actif vs HC
	pre-treatment	post-treatment	pre-treatment	post-treatment		p value	p value
Accuracy 0-back	98.8 (+/−3.5)	98.8 (+/−2.3)	99.3 (+/−1.2)	99 (+/−2.4)	99.5 (+/−0.8)	0.6; 0.5	0.6; 0.6
RT 0-back	436.9 (+/−52.7)	440.4 (+/−79.4)	493.6 (+/−165.6)	501.5 (+/−159.8)	446.2 (+/−117.5)	0.9; 0.9	0.5; 0.4
Accuracy 1-back	98 (+/−2.4)	98.3 (+/−3.1)	95.5 (+/−6)	97.3 (+/−2.2)	96.3 (+/−4.3)	0.4; 0.4	0.8; 0.6
RT 1-back	719.4 (+/−168.5)	867 (+/−249.8)	801.2 (+/−165.5)	703.3 (+/−323)	671.3 (+/−182.8)	0.6; 0.1	0.2; 0.8
Accuracy 2-back	93.2 (+/−0.3)	91.1 (+/−7.3)	89.7 (+/−7.9)	89.3 (+/−7.2)	93.6 (+/−5.3)	0.9; 0.5	0.3; 0.2
RT 2-back	838.5 (+/−361.3)	1184.9 (+/−443.2)	1114.4 (+/−219)	909.7 (+/−278.7)	806.8 (+/−274.2)	0.9; 0.7	**0.03**; 0.5
Accuracy 3-back	83.1 (+/−11)	86.2 (+/−6.6)	75.2 (+/−9.2)	78.7 (+/−11.6)	89 (+/−5.2)	0.2; 0.4	**0.002**; **0.04**
RT 3-back	1014.5 (+/−337.5)	1349.9 (+/−220.3)	1359.5 (+/−211.5)	1083.2 (+/−357.7)	992.1 (+/−239.6)	0.9; **0.01**	**0.008**; 0.6

Response times (RT) were more sensitive to group and treatment differences than was accuracy: a three-way ANOVA with repeated measures showed a significant group × treatment × n-back interaction (p = 0.02). Subsequent one-way ANOVAs showed that active CPAP group performed significantly worse than HC on 2 (p = 0.03) and 3-back (p = 0.008) at baseline, whereas after treatment there was no difference between active CPAP and HC at 2 (p = 0.47) and 3-back (p = 0.6). In comparison sham CPAP group showed no difference with HC at baseline, but performed significantly worse than HC after sham treatment on 3-back (p = 0.01).

There was no significant difference in performance for the other levels of task load between the two patients' groups or when looking at each group response compared to HC (Table 2).

In order to exclude the potential role of fatigue on brain activation and performance, we looked for a relation between RT during the 0-back epochs throughout both sessions of the n-back task with task duration. There was no correlation between RT and task duration for either session.

### fMRI

The activation/deactivation patterns in our HC group mobilized cerebral regions previously described as involved during performance of WM tasks [19]. Both activation and deactivation increased with increasing task difficulty in all groups, suggesting that subjects maintained their effort at all load levels.

TPN activation and DMN deactivation refer to increased and decreased cerebral activity respectively in a given brain region in one of the three task loads (1,2 and 3-back) as compared to 0-back (baseline).

All reported results represent significant differences in activation/deactivation at p<0.05, corrected for multiple comparisons.

#### 1. Paired T-tests in OSAS patients

In the therapeutic CPAP group, at baseline, compared to the 2 months post treatment condition, no location of the TPN had evidence of higher activation in any of the 3 contrasts (1vs0, 2vs0 and 3vs0-back). On the contrary, after 2 months of treatment there were significantly higher activations in the bilateral frontal regions of the TPN (middle frontal gyri (BA 6)) in all contrasts and in left precuneus (BA 7) in the 3vs0-back contrast.

In the DMN regions, there was significantly higher deactivation in the 3vs0-back contrast in the anterior midline hub of the DMN (ACC (BA 32) extending to medial frontal gyrus) at baseline as compared to after CPAP treatment (Table 3A).

**Table 3:** Therapeutic and sham-CPAP effects: cerebral regions showing significant task-related activation and deactivation in TPN and DMN regions on paired t-test in 1vs0, 2vs0 and 3vs0 contrasts in A) the active CPAP group and B) sham-CPAP group (MNI coordinates, corrected for multiple comparisons, p = 0.05).

	Region	Brodman	x	y	z	Zscore	cluster size
A. CPAP group							
TPN							
Baseline>post-ttt	none						
post-ttt>baseline							
1>0	R sup/middle	BA 6,8	34	18	56	3.08	84
	frontal						
	L middle/sup	BA 6,8	−30	2	68	3.71	64
	frontal						
2>0	L middle frontal/	BA 6	−24	−6	62	4.3	507
	precentral						
	R middle frontal	BA 6	40	2	52	3.5	80
	R sup frontal	BA 6	18	4	54	4.05	95
3>0	L middle frontal	BA 6	−26	−8	62	3.69	167
	R middle frontal	BA 6	28	2	66	3.36	84
	L precuneus	BA 7	−8	−72	44	3.36	84
DMN							
Baseline>post-ttt							
0>1	none						
0>2	none						
0>3	L ACC/medial	BA 32	−6	46	0	2.9	71
	frontal						
post-ttt>baseline	none						
							
B. sham-CPAP group							
TPN							
Baseline>post-ttt	none						
post-ttt>baseline	none						
DMN							
Baseline>post-ttt							
0>1	none						
0>2	L precentral	BA 4	−64	−10	30	4.14	81
0>3	none						
post-ttt>baseline							
0>1	none						
0>2	L middle/sup	BA 21,38	−38	6	−40	3.43	147
	temporal						
0>3	none						

In the sham CPAP group we found no significant difference in TPN activation between baseline as compared to 2 months of sham CPAP treatment.

In the DMN regions, there was significantly higher deactivation in the 2vs0-back contrast in the left middle temporal gyrus (BA 21) extending into the temporal pole (BA 38) after sham-CPAP treatment as compared to baseline (Table 3B).

#### 2. Treatment group versus scan session interaction

There was no significant interaction in the activation of either TPN or DMN regions in any of the examined contrast.

#### 3. Comparison with Healthy Controls (3vs0 contrast)

As behavioral differences between the two patient' groups and HC were only observed in the 3vs0 contrast, we only report the differences in cerebral activation between HC and OSAS patients for that contrast (Table 4; tables of differences for 1vs0 and 2vs0 contrasts are available upon request from corresponding author).

**Table 4:** CPAP and sham-CPAP groups compared to HC: cerebral regions showing significant task-related activation and deactivation in TPN and DMN regions in 1vs0, 2vs0 and 3vs0 contrasts in active and sham-CPAP patient' groups as compared to HC (MNI coordinates, corrected for multiple comparisons, p = 0.05).

	Region	Brodman	x	y	z	Zscore	Cluster size
Baseline							
TPN							
CPAP group>HC	None						
HC>CPAP group	R frontal	BA 9	42	4	28	3.13	89
	opercule						
	L middle	BA 6	−30	−4	64	3.66	74
	Frontal						
sham group >HC	none						
HC>sham group	none						
DMN							
CPAP group >HC	L cuneus/	BA 19	−28	−80	8	2.88	101
	middle occipital						
HC>CPAP group	none						
sham group >HC	L middle occipital	BA 19	−28	−78	−6	4.16	65
HC>sham group	none						
Post-treatment							
TPN							
CPAP group>HC	L inf parietal	BA 40	−36	−54	50	3.81	100
	lobule						
HC>CPAP group	none						
sham group >HC	none						
HC>sham group	R inf frontal/	BA 47,13	34	18	−2	3.28	134
	insula						
	R precentral/	BA 44	58	10	6	3.64	62
	inf frontal						
	R inf frontal	BA 9	38	4	28	2.95	42
DMN							
CPAP group>HC	L lingual	BA 18	−24	−72	−10	3.12	99
HC>CPAP group	none						
sham group >HC	none						
HC>sham group	none						

Compared to HC, at baseline, the CPAP group had significantly less TPN activation in the left middle frontal gyrus (BA 6), which normalized after 2 months of CPAP treatment.

In the DMN regions, at baseline OSAS patients had higher deactivation in the 3 vs 0-back contrast as compared to HC in the left cuneus (BA 18), which normalized after treatment.

After CPAP treatment, OSA patients also displayed higher activation within TPN's left inferior parietal lobule (BA 40) and DMN's left lingual gyrus (BA 18).

In the sham-CPAP group, there was no difference in TPN regions' activation between patients and HC at baseline. After sham treatment, the sham-CPAP group lost TPN activation in the right frontal opercular region partially corresponding to the ventral frontal cortex component of the ventral frontal cortical network described by Corbetta and colleagues [14].

Higher deactivation was observed at baseline in the left middle occipital region in OSAS patients as compared to HC. There was no difference in DMN regions' deactivation between patients and HC after sham-CPAP treatment.

**Figure 2:**
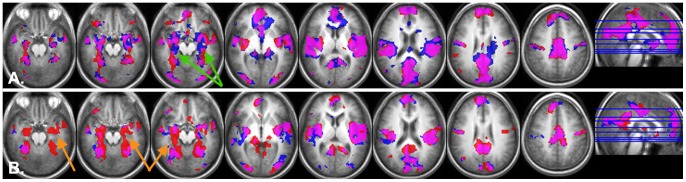
Brain regions showing significant task-related deactivation in the 0vs3-back contrast in A) 1) CPAP group at baseline AND HC (red); 2) CPAP group post-treatment AND HC (blue; purple indicates overlap between 1 and 2); and in B) sham-CPAP group at baseline AND HC (red); 2) sham-CPAP group post-treatment AND HC (blue; purple indicates overlap between 1 and 2). Green arrows indicate increased deactivation in the tDMN after CPAP treatment (regions in blue), orange arrows indicate decreased deactivation in the tDMN after sham-CPAP treatment (regions in red) (neurological presentation, p<0.05, FDR corrected for multiple comparisons).

Inclusive masking analysis showed little differences between OSAS patients (before and after treatment) and HC (at baseline) in TPN activation (3 vs 0-back contrast), however opposite evolution was present in the CPAP and sham-CPAP groups in the DMN deactivation (0 vs 3-back contrast) in the medial temporal regions (hippocampi and parahippocampal gyri). CPAP group gained some deactivation after treatment, whereas the sham-CPAP group lost DMN deactivation after 2 months of sham-CPAP (Fig. 2). Thus, the masking analysis allowed us a finer insight into changes in cerebral deactivation within the DMN that are not yet apparent in the interaction analysis as addressed with two-sample t-tests or a flexible factorial analysis at p<0.05 corrected for multiple comparisons.

Globally, the sham group had fewer differences with HC both before and after the sham-condition than the active CPAP group, likely due to initial differences in OSAS severity.

To summarize, the sham and active-CPAP groups followed opposite trends of activation and deactivation across task levels after treatment, particularly evident at 3 vs 0 contrast, which is in line with behavioral performance results.

Active-CPAP group exhibited higher activation in a number of TPN regions after treatment as compared to baseline in all three contrasts (and no region had higher activation in any contrast at baseline as compared to after treatment), but the sham-CPAP group showed no regions of higher activation after 2 months of sham CPAP treatment in any contrast, and in the 3 vs 0 contrast this group exhibited even a loss of activation in the Ventral Frontal Cortex (VFC). Within the DMN, inclusive masking analysis revealed a similarly opposite evolution between CPAP and sham-CPAP groups with an increase of DMN deactivation in the medial temporal regions (hippocampi/parahippocamapal gyri) after CPAP treatment and a decrease of DMN deactivation after sham-CPAP treatment (Fig. 2).

The sham-CPAP group showed no difference with HC in the TPN regions at baseline as opposed to the active CPAP group, reflecting, we believe, the OSAS severity differences between the groups at baseline. At baseline, both groups had more DMN deactivation than HC, which, in light of the preserved behavioral performance may be interpreted as a compensatory activity in the DMN. After 2 months of sham CPAP however, the TPN deficits increased, particularly in the VFC and the compensatory DMN deactivation decreased, in parallel with a decrease in behavioral results.

#### 4. Multiple regression analysis results (OSAS patients only)

We found a significant positive correlation between the duration of nocturnal hypoxemia and cerebral activation within DMN in the left temporal pole, right parahippocampal gyrus, bilateral fusiform gyri, and left posterior cingulate cortex. We found no positive correlation between the duration of nocturnal hypoxemia and cerebral activation within TPN.

**Figure 3:**
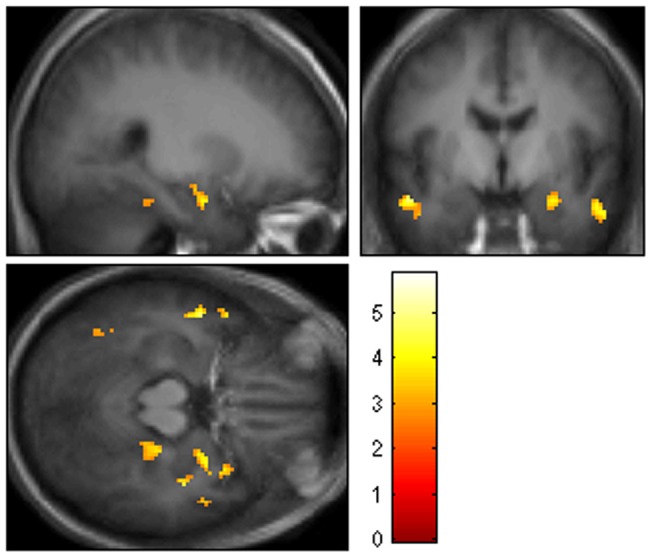
Brain regions showing significant positive correlation between the duration of nocturnal hypoxemia and effects of task load in DMN (ie, regions where increased duration of nocturnal hypoxemia correlates with less effective deactivation of DMN regions with increasing task load; MNI coordinates, corrected for multiple comparisons, p = 0.05, neurological presentation).

Significant negative correlation was exhibited between the duration of nocturnal hypoxemia and cerebral activation within a) DMN in the right cingulate (BA 31/24) and right precentral (BA 6) gyri; b) TPN in the right inferior frontal gyrus (BA 9) (Fig. 3, Table 5).

**Table 5:** Effects of nocturnal hypoxemia on cerebral activation in OSAS patients: cerebral regions showing significant positive correlation between the duration of nocturnal hypoxemia and effects of task load in DMN (ie, regions where increased duration of nocturnal hypoxemia correlates with less effective deactivation of DMN regions with increasing task load; MNI coordinates, corrected for multiple comparisons, p = 0.05).

	Region	Brodman	x	y	z	Zscore	cluster size
Positive correlation							
with hypoxemia	L middle/sup	BA 21, 38	−52	−4	−20	3.62	167
in DMN	temporal						
	R parahippocampal/		32	8	−14	4.1	137
	sup temporal/						
	amygdala						
	L fusiform		−44	−58	−16	3.48	60
	R parahippocampal/	BA 36	24	−30	−18	3.15	61
	fusiform						
	R fusiform/middle temporal	BA 20/21	56	−4	−28	3.28	120
	L PCC/precuneus	BA 31	−20	−54	18	3.31	399
in TPN	none						
Negative correlation	R precentral	BA 6	42	−16	34	3.43	76
with hypoxemia	R cingulate	BA 31/24	6	−22	44	3.17	73
in DMN							
in TPN	R inf frontal	BA 9/8	50	14	38	3.37	42

There was no significant correlation between AHI and cerebral activation within either DMN or TPN regions.

Several brain regions showed significant correlation with both AHI and duration of nocturnal desaturation (bilateral cingulate gyri, transverse gyri, inferior parietal lobules, middle frontal gyri (BA 10), supplementary motor areas, right dlPFC, right fusiform gyrus). However, due to collinearity between those two variables, the direction of their individual contributions could not be determined for those regions.

## Discussion

To our knowledge, this is the first study specifically examining the effects of CPAP on TPN and DMN networks of OSAS patients and comparing results to those observed in HC and in patients with sham CPAP exposure, using fMRI and a working-memory task with 4 levels of increasing load.

The main finding of our study was that active and sham CPAP have opposite effects on behavioral performance and cerebral activation, particularly apparent at the limit-of-capacity task level (3-back).

Most differences between our HC and untreated OSAS patients are found in the deactivation of the temporal regions of the DMN (tDMN) and in TPN activation of bilateral frontal eye fields and of the ventral frontal cortex (VFC) of the right-hemisphere ventral frontal network. On the contrary, TPN activation within the bilateral fronto-parietal network, including dlPFC, was comparatively relatively preserved. Differences are more pronounced in the 3 vs 0-back contrast, where both patient groups performed significantly worse than HC.

After 2 months of treatment, active- and sham-CPAP groups showed opposite trends of cerebral activation: this is particularly apparent at the 3-back task level, suggesting a positive effect of CPAP on the active-treatment group while the sham-CPAP group seems to sustain additional effects of untreated OSAS, or unreported effects of sham-CPAP exposure. An alternative interpretation of our results obtained on the sham group, is that sham-CPAP per se has a negative impact as it may lead to more mouth-breathing and sleep disruption. We could not directly assess the effects of sham-CPAP on the sleep continuity in our patients, but the significant worsening of RTs and decreased TPN activation together with decreased DMN deactivation in the temporal regions at the 3-back after 2 months of sub-therapeutic exposure in the sham group reflects a rather faster evolution than what one would expect of untreated OSAS-related effects.

In a previous report we found a positive correlation between the duration of nocturnal desaturation and cerebral activation in the medial and anterior regions of temporal lobes of untreated OSAS patients. We interpreted these findings as resulting from the particular vulnerability of the temporal structures to hypoxic stress [19]. In the present study we improved upon our previous analysis by examining the effects of AHI and nocturnal hypoxemia on cerebral activation specifically within TPN and DMN, which revealed an even more extensive impact of hypoxemia on temporal lobe structures, while AHI effects were comparatively modest (Fig. 3, Table 5). Our findings of opposite response in deactivation of the temporal brain regions after 2 months of active and sham-CPAP treatment support our initial hypothesis: that OSAS has a negative impact particularly on the temporal lobe part of the DMN, possibly via the effects of nocturnal hypoxemia. Our results are corroborated by the findings of the recent multicentric APPLES study showing hypoxemia as the major OSAS-related factor to correlate with behavioral cognitive deficit in OSAS patients [26] and by the fact that overall, the most consistent findings in structural neuroimaging studies of OSAS patients are signs of hippocampal injury (atrophy or metabolic anomaly) [27], [28], [29], [30]. Similarly, in a recent study, O'Donghue et al have found metabolic abnormalities in the hippocampus of OSAS patients comparable in magnitude to changes found in Alzheimer's disease [31]. Those changes were no longer detectable after 6 months of treatment, suggesting that the hippocampus, even if sensitive to OSAS-related injury, has the potential for recovery with CPAP treatment.

Defective deactivation of DMN regions is associated with worse behavioral performance in a number of conditions associated with cognitive impairment and sleep disturbances (normal aging, Parkinson disease, schizophrenia, ADHD), suggesting that inability to reallocate neuronal resources and to suppress activity in neighboring regions plays a role in cognitive impairment [32], [33], [34], [35]. Disruption of task-related deactivation within the midline regions of DMN is seen after acute sleep deprivation [36]; and slower reaction times on a psychomotor vigilance task are also associated with increased activation in midline DMN regions after total sleep deprivation [37], suggesting that sleep deprivation has a direct impact on DMN.

Sleep deprivation (SD) induces a decrease of cerebral activation in the fronto-parietal and ACC regions of the TPN and reduces DMN deactivation during WM tasks [38], [39], [40], [41]. The improvement that we observed after CPAP treatment (in cerebral task-related activation-deactivation and behavioral parameters), associated to partial recovery of task-related deactivation in tDMN regions and in midline core DMN regions, likely reflects two aspects of CPAP action: elimination of episodes of nocturnal hypoxemia and decrease in sleep fragmentation with subsequent improvement of vigilance levels. The “improvement of alertness” effect.is also supported by the higher activation shown by HC compared to OSAS patients in the bilateral anterior insular/inferior frontal regions (BA 47/13)(which constitute the “alerting” node of the TPN [42], [43]), as well as in the right VFC [14] and by the accentuation of these differences after sham CPAP treatment.

Corbetta and colleagues have proposed that the right hemisphere-lateralized frontal network's function is to detect behaviorally relevant stimuli and works as an alerting mechanism or “circuit-breaker” for the bilateral fronto-parietal network when these stimuli are detected outside of the focus of cognitive processing [14]. The frontal component of this network is affected in OSAS patients and may explain further the increased accident risk reported in these patients: in addition to impaired vigilance OSAS patients may suffer from an impaired ability to detect behaviorally relevant stimuli outside of their attention set and in their ability to shift attention in order to make an adequate response.

Two other studies have investigated the effect of CPAP on cerebral activation of OSAS patients. Thomas et al. (2005) have found no recovery of dlPFC activation on a verbal 2-back WM task in 6 OSAS patients after CPAP treatment, although there was some increase of parietal activity [7]. Since their patient group performed significantly worse than HC at baseline and did not improve after treatment, the lack of cerebral activation changes in the dlPFC was interpreted as an indication of a more permanent cerebral damage. However, no information was reported on the deactivation pattern.

More recently Castronovo et al. (2009) examined the effect of CPAP on cerebral activation on a verbal 1- and 2-back WM task in 17 OSAS patients [12]. Greater activation with WM load was reported in a number of prefrontal regions, bilateral precuneus, left putamen, left hippocampus in never treated OSAS patients as compared to HC and was interpreted as a compensatory over-recruitment. If more extensive activation in the PFC is indeed suggestive of compensatory recruitment of the TPN, more important activation with WM load in the hippocampus can be better explained by defective deactivation with WM load. Together with their finding of lesser activation with WM load in bilateral hippocampi after CPAP treatment, these observations are consistent with our findings of defective DMN functioning in OSAS. Considering that OSAS patients of Castronovo et al performed similarly to HC on all task levels, their patient population can be more directly compared with ours, who did not reveal behavioral differences with HC at 1 and 2-back levels. It appears that in this subset of OSAS patients as opposed to subjects with behavioral deficit studied by Thomas et al, dlPFC function is still intact whereas hippocampal deficits are already present, even though partially reversible with treatment. But, an important methodological point, only the inclusion of limit-of-capacity 3-back level allowed us to observe behavioral differences between patients and HC, as well as the interaction in cerebral activation/deactivation between the two patient' groups (active and sham CPAP).

Our study has several limitations: the fact that we had no untreated patient control group does not allow us to firmly tease apart the possible negative effects of sham-CPAP and the effects of the natural evolution of untreated OSAS. Similarly, the fact that HC have undergone only one fMRI session does not allow complete control of possible learning effects when comparing them to OSAS groups after treatment. In order to minimize this potential bias, we have trained all our subjects twice before the baseline scan at 1-week interval, until maximal performance could be achieved (>75% for 3-back). Moreover, when comparing pre ant post-treatment changes in brain activity, we only report within-subjects contrasts. Finally, the fact that the 2 patient groups exhibited opposite trends of activation and deactivation also suggests that any potential learning effects after 2 months were minimal.

The relatively small sizes of our patient' groups allowed for differences in BMI and behavioral performance at the intermediate (2-back) and maximal WM loads (3-back) between sham and active CPAP groups at baseline despite randomization. It is increasingly recognized that excess body weight is associated with brain structural and functional alterations [44], a higher risk of developing dementia in later life [45], as well as cognitive dysfunction in otherwise healthy adults or after controlling for BP, age, and diabetes [45], [46], [47]. Therefore higher BMI in the active CPAP group might account for failure of this group to completely normalize their behavioral performance at the 3-back level after treatment. However, this did not obscure the opposite evolution of the two groups and allowed a further insight into the differences between HC and OSAS patients with different degrees of performance impairment.

Finally, we used relatively short treatment duration of 2 months and it is possible that further improvements in behavioral performance and cerebral activation/deactivation patterns could be seen with longer treatment durations, such as 6 months.
